# Optimizing Patient Management and Adherence for Children Receiving Growth Hormone

**DOI:** 10.3389/fendo.2017.00313

**Published:** 2017-11-20

**Authors:** Carlo L. Acerini, Katarzyna Wac, Peter Bang, Dagmar Lehwalder

**Affiliations:** ^1^Department of Paediatrics, University of Cambridge, Cambridge, United Kingdom; ^2^Quality of Life Technologies Laboratory, University of Geneva, Geneva, Switzerland; ^3^Department of Clinical and Experimental Medicine, Linköping University, Linköping, Sweden; ^4^Global Medical Affairs, Merck KGaA, Darmstadt, Germany

**Keywords:** pediatric growth hormone response, adherence, growth hormone administration devices, digital health, personalized medicine, endocrinology

## Abstract

Poor adherence with growth hormone (GH) therapy has been associated with worse clinical outcomes, which in children relates specifically to their linear growth and loss of quality of life. The “360° GH in Europe” meeting, held in Lisbon, Portugal, in June 2016 and funded by Merck KGaA (Germany), examined many aspects of GH diseases. The three sessions, entitled “*Short Stature Diagnosis and Referral*,” “*Optimizing Patient Management*,” and “*Managing Transition*,” each benefited from three guest speaker presentations, followed by an open discussion and are reported as a manuscript, authored by the speakers. Reported here is a summary of the proceedings of the second session, which reviewed the determinants of GH therapy response, factors affecting GH therapy adherence and the development of innovative technologies to improve GH treatment in children. Response to GH therapy varies widely, particularly in regard to the underlying diagnosis, although there is little consensus on the definition of a poor response. If the growth response is seen to be less than expected, the possible reasons should be discussed with patients and their parents, including compliance with the therapy regimen. Understanding and addressing the multiple factors that influence adherence, in order to optimize GH therapy, requires a multi-disciplinary approach. Because therapy continues over many years, various healthcare professionals will be involved at different periods of the patient’s journey. The role of the injection device for GH therapy, frequent monitoring of response, and patient support are all important for maintaining adherence. New injection devices are incorporating electronic technologies for automated monitoring and recording of clinically relevant information on injections. Study results are indicating that such devices can at least maintain GH adherence; however, acceptance of novel devices needs to be assessed and there remains an on-going need for innovations.

## Introduction

When growth hormone (GH) is administered to children with short stature, the growth response to treatment may vary widely between patients. This is partly related to the underlying cause of the short stature, but there are also other factors that are involved, including poor adherence to the therapy regimen itself. There are multiple causes for treatment non-adherence, some of which have been addressed by adapting and improving the design of GH administration injection devices. However, resolving issues of GH non-adherence requires a much broader multi-disciplinary approach, which needs to be able to change as the young patient transitions through the different stages in their life. Monitoring of treatment adherence is, therefore, crucial and can be increasingly achieved by the application of new technologies, including those directly integrated into the injection device itself. This report from the second session of the 360° GH in Europe meeting reviews the determinants of GH therapy response, the factors affecting GH therapy adherence and the innovative technologies that are being developed to improve these issues. The reports on the first and third sessions, on the diagnosis and referral of patients with short stature and on the transition of GH-treated patients from pediatric to adult care, are published in accompanying articles (1, 2).

## GH Responders Versus Non-Responders—Identification and Management

In pediatric patients with short stature, one of the main determinants of response to GH treatment is the underlying diagnosis. Plotting GH secretion from stimulation tests versus sensitivity, defined as change in height standard deviation score (SDS) over time, shows a continuum of response (3, 4). This ranges from severe GH deficiency, with low secretion and good sensitivity, through to severe primary IGF-I deficiency, with good secretion and low sensitivity. However, the means of determining GH secretion remain equivocal, and sensitivity cannot be evaluated prior to treatment, but only at some time after GH initiation; it is usually derived from height change at 1 year. As a result, there is currently no consensus on how to separate groups of responders and non-responders to GH treatment, with wide variation seen within as well as between each of the diagnostic groups (3).

Various measures have been suggested to enable definition of a poor response to GH. A 1-year change in height SDS of <0.3 is not sufficiently different from the normal growth rate and is not a sufficiently accurate definition of a poor response. A cutoff height SDS change of 0.5 is more specific, corresponding to about 1.5 cm above normal annual growth at 2 years of age and about 3 cm above normal at 10 years of age. Whatever definition is used though, there remain a high number of patients defined as poor responders in all diagnostic groups, ranging from 10% to about 60% of GH-treated patients (3).

In a large US study, measurement of height velocity in the first year of treatment showed a marked difference according to age, with decreasing change in mean height velocity in patients who were older at GH initiation (5). The results indicated that a first-year height velocity more than 1 SD below the mean for age was an acceptable cutoff for a poor response. Height velocity data from patients with GH deficiency in the US study were shown to be in accord with results in the Pfizer International Growth (KIGS) database (6). However, height velocity minus 1 SD in the KIGS data corresponded to minus 2 SD in the US study because the GH deficiency was more severe for the US patients and the GH dose used for GH deficiency in the US is higher than in Europe.

It should be remembered though that not only GH sensitivity may differ in a patient, but also IGF sensitivity may vary. In a study that tried to remove the variation in GH response by adjusting the dose to achieve a target IGF-I SDS of either 0 or 2, it was found that there were still wide differences in 1-year change in height SDS (7). Data for differing diagnoses were suggested to indicate varying degrees of both GH and IGF-I insensitivity; thus, the authors concluded that patients with different diagnoses may require different management strategies to optimize growth.

There are models that have been used to predict growth response prior to GH initiation (8). However, in patients with a predicted 1-year change in height SDS of <0.5, assessment of the observed change resulted in a large number of patients who had an actual height SDS change >0.5 (3). This suggests that these prediction models cannot be used to accurately predict patients with poor response prior to treatment. Nevertheless, if the observed response at 1 year for an individual patient is below the predicted response, this can be used to discuss the possible reasons with patients and their parents. There may be several reasons, such as an incorrect diagnosis of cause of short stature, occurrence of concomitant diseases, or unanticipated GH insensitivity (6). Alternatively, poor adherence to a treatment regimen may be identified in a patient, and different management may be required in order to try to overcome this.

## Adherence to GH Treatment—A Practical Approach

Poor adherence with GH therapy has been demonstrated to be associated with worse clinical outcomes, specifically impaired linear growth in children (9). Treatment failure and loss of quality of life may result from non-adherence to GH regimens (10). However, the evidence base for assessing adherence to GH therapy remains rather weak, with relatively few studies and wide variation in their quality and definitions. A recent systematic review of published studies found estimates for pediatric non-adherence ranging from 5 to 82% (11), although it is generally evident that most patients are, at times, non-adherent to some degree. A number of different factors have been identified that are known to influence treatment adherence in general, including those relating to the patient, their underlying disease, and their understanding of the disease and the need for treatment, as well the healthcare professionals and the quality of the healthcare system supporting them. The type, nature, and route of administration of the treatment are also important factors that influence treatment adherence rates (11).

Understanding these factors may suggest ways in which GH therapy adherence could be improved. The treatment modality is crucial in affecting growth, and the efficacy, safety, and frequency of administration will directly affect adherence. The healthcare system has to be efficient with follow-up and monitoring and be able to provide well-coordinated clinical practices. The physicians and other personnel involved in therapy need to have a good knowledge base of both the disease and the treatment options; this empowers them to help both the patients and their families. Underlying all of this is involvement of the patients and families in decision making; providing them with sufficient knowledge of the condition and the benefits/risks of a therapy option is crucial for maintaining compliance of pediatric patients (12).

A good knowledge base allows optimization of therapy and this should be provided both at the introduction and during maintenance phases of a specified therapy. Information must be provided at the appropriate times, the expectations of everyone involved should be identified and understood, and concerns should be addressed as soon as they arise. Patients and families should be included in decision making, with interventions tailored to help them, in order to provide a consensus approach to therapy initiation and maintenance and achieve better outcomes (10). Monitoring of adherence is also important to allow non-adherence to be tracked and assessed. Knowledge and understanding of the condition and treatments is particularly important as the young patient gets older, particularly as they transition from childhood to adolescence. Changes in requirements should be identified as soon as possible, enabling problems to be resolved more efficiently. Where adherence behavior becomes aberrant, a psychological approach enables recognition and resolving of issues that occur. Various models are available to address adherence, such as the Capability, Opportunity, Modification Behavior (COM-B) model, which has been applied in other therapy areas (13, 14).

It must be emphasized that optimizing GH therapy adherence is not the sole responsibility of the patient or their family, nor of the prescribing pediatrician/physician. There needs to be a multi-disciplinary approach, involving a number of different healthcare professionals and allied agencies. While the pediatric endocrinologist and specialist endocrine nurses are central to this supportive role, pharmacists can also ensure timely and efficient drug dispensing, and clinical psychologists and play therapists may help with issues such as needle or injection phobia.

During a young patient’s treatment journey, multiple opportunities exist for reviewing adherence and resolving any issues that arise. This may start at the time of diagnosis and with the initiation of GH treatment, and should continue at all routine follow-up clinic visits thereafter. There are also specific developmental “milestone” opportunities where adherence may be assessed, including at onset of puberty and transition to adolescence, moving from junior to high school, at completion of growth and transition to care as a young adult, and at the start of higher education (15). Where poor adherence is suspected, specific patient support packages can be put in place. This might include provision of further counseling, education, and training. Young patients may also change their preferences to a particular GH administration device, and, when this occurs, a change in GH injection device may be required. The application of incentive or reward systems may be helpful when dealing with younger patients, including the use of calendar/reminder systems and other token reinforcement strategies. Finally, patient/family support groups can also provide invaluable advice and support regarding GH therapy.

## Research and Technology to Help Achieve Patient Outcomes

Almost 80% of European inhabitants now have access to the Internet (16), which has a huge economic impact, affects daily life and is enabling new trends in digital health. Miniaturized, wearable personal devices are becoming more common on a growing scale, with US sales of wearable bands increasing from 17 million in 2014 to 23 million in 2015, and estimated to be 45 million in 2017 (17).

Technological innovations have already been shown to be effective in several other areas of medicine. A computer-based service was associated with good compliance in patients with hypertension in 1994 (18) and, more recently, internet-based computer systems have improved adherence with diabetes medications (19) and psychotherapies (20). Mobile phone system reminders have been used to improve adherence with therapies for diabetes (21, 22), HIV (23), and malaria (24). Smart watches and smart pill packages are also now being used for reminders and monitoring of medication intake, which also improves adherence (25). For asthma management in children, an inhaler with audiovisual reminders and electronic monitoring increased corticosteroid adherence to 84% compared with 30% in a control group, and was associated with improved asthma morbidity score (26).

With regard to GH therapy, the role of the injection device, frequent monitoring, and patient support are very important for maintaining adherence, and there is a need for automatic monitoring and recording of information on injections (10, 27, 28). There are already technological improvements being made in devices for GH administration. A questionnaire study in patients and physicians concluded that new GH administration devices needed to be reliable, simple to use, and cause minimal pain (29). Discussion with the patients and physicians regarding four different devices indicated that an electronic device was preferred to an existing automated device, a needle-free device or a disposable injection device (29).

New injection devices are incorporating electronic monitoring systems that enable the physician to identify time and dose of administration. The only such device for GH treatment, with published information at present, is the easypod™, which is an electronic auto-injector for administration of GH (Saizen^®^, Merck KGaA, Darmstadt, Germany) that was launched in 2007. This is now approved for use in more than 40 countries and has shown high acceptance by both children and their parents (30). During testing, users were reported to have a good overall impression, with a majority expressing a preference to continue to use it (30). After the dose is pre-set by a physician or nurse, patients can then set the depth, speed, and duration of injections (31). The device can monitor times and doses administered, with number of doses remaining being displayed to allow patients to better manage their schedule. The data can be automatically transmitted to the physician, who is able to assess adherence; however, patients retain ownership of their data. If adherence appears to decrease at any time, the physician can contact the patient and try to address any issues before GH effectiveness is lost. In a 3-month study in 824 children, adherence was considered good and was significantly better in treatment-naïve children just starting GH than in treatment-experienced children (32). Interim data from a large observational study has also indicated a high rate of adherence with GH therapy using the device, with medians of 95–97% in Canada, France, and Nordic countries at 9 months (33).

Technological innovations can only help if they are accepted and used by patients and their caregivers. A literature review suggested that user acceptance of telemedicine services among physicians and patients accounted for up to 37% of the success of telemedicine services (34): technology accounted for 29%, organizational aspects for 13%, policy and legislation for 11%, and financing for 10%. Knowledge of factors that will enable an increase in this acceptance rate for GH treatment are currently lacking; solutions to address them include understanding the expectations of prescribers and patients for the technology, evidence for new GH treatment behavior changes that are enabled by new technologies and verification of whether prescribers need a behavior change in order to leverage innovative solutions.

## Discussion Session

In order to overcome variation in the growth response to GH treatment, it has been suggested that IGF-I level could be used routinely to manage the administered dose. There remain safety issues with increasing the GH dose though, and patients should only be treated with the approved doses. IGF-I concentration can change reasonably quickly and a savvy adolescent may take injections only for a short time to increase IGF-I just before a clinic visit, to disguise non-adherence. In GH-deficient patients, normal levels of IGF-I and IGFBP-3 are reached in 4–7 days after starting GH therapy, so the approach in such possible cases may be to monitor the levels more frequently, rather than just at the routine visits, in order to judge adherence.

Continuous infusion of GH has also been suggested as a means to improve response, but the only published study on continuous infusion found no difference in response, although IGF-I increased to a significantly greater extent compared with once-daily injection (35). Investigational long-acting formulations have been proposed to have the potential to increase adherence (36). A number of different long-acting GH products have been investigated, although few have shown sufficient efficacy and safety, and none are currently marketed except for one in China only (37). None of the published studies of long-acting GH have shown an increase in adherence. However, novel devices that collect information regarding adherence may help to understand whether variation in response to GH relates to adherence or to other factors. Further adherence data that are being collected using electronic injection devices may help healthcare practitioners in the effective management of patients (38). In particular, an automated messaging function in a device can alert healthcare workers of reduced adherence of a patient and can provide an indication of different levels of importance.

Adherence could possibly be increased for some patients by reducing the number of daily injections (e.g., from 7 to 6 per week). Flexibility is considered a reasonable approach, but no studies have established whether the dose should be adjusted with fewer injections. While the outcome may be reduced with constantly missed doses, it is difficult to predict what outcome should be expected. However, response is diagnosis-specific, and reducing doses could possibly be less important for patients with short stature due to severe GH deficiency than for patients born SGA.

Whether patients get a choice on formulations and devices, or if physicians give only one choice, should be considered when assessing compliance with GH treatment (9). In the UK, patients are always given a complete choice of GH brands and devices; in contrast, it was noted that in Portugal some hospitals offer only one choice, while others offer all brands. In the United States, device preference for GH administration is at the discretion of the insurance company; demonstration of increased adherence according to the device used may provide additional information for insurance companies to allow alternative options for clients to choose their preferred device.

Frequency of alerts and the type of patients that should be closely monitored for adherence need to be established. The urgency of alerts is unlikely to be considered the same by patients as by healthcare providers, and physicians need to establish what clinical outcomes are desired. Some patients and their families may resent too much intrusion and may consider feedback on adherence to be over frequent because they do not consider the situation to be urgent. Patients and healthcare workers who are technically knowledgeable will accept adherence monitoring, but the clinical value still needs to be assessed. In adolescent patients with diabetes, for instance, text messaging has been shown to have little impact on adherence or outcome (39). Nevertheless, approaches such as the COM-B model could be effective in overcoming issues relating to poor adherence, and techniques to change behavior have been shown to be efficacious (14, 40).

Expectations of pediatric patients and their parents need to be assessed. There is a need to increase understanding of the management of expectations, from both clinical and psychological viewpoints. Expectations need to be established right from the start in order to get long-term adherence. When considered purely as height growth, there is a physiological decrease in apparent response to GH treatment over time; therefore, information about other benefits, such as metabolic effects and bone strength, should be conveyed as patients enter adolescence.

Guidelines on when to stop GH treatment also need to be identified. Psychological factors influencing the patients and their families need to be considered to see if these may be causing problems of adherence. If treatment is continued long-term, and particularly as adolescent patients enter transition to adult care, then adherence should continue to be monitored over time and novel techniques and devices can help with this.

## Author Contributions

All authors critically revised the current work for important intellectual content and gave final approval of the version of the publication to be published. All authors have agreed to be accountable for all aspects of the work in ensuring that questions related to the accuracy or integrity of any part of it are appropriately investigated and resolved.

## Conflict of Interest Statement

CA reports personal fees from Ferring, personal fees from Merck KGaA, Darmstadt, Germany, personal fees and non-financial support from Novo Nordisk outside the submitted work. KW reports personal fees from Merck KGaA, Darmstadt, Germany, for participation in the 2016 growth hormone congress and personal fees from Novartis outside of the submitted work. PB reports personal fees from Merck KGaA, Darmstadt, Germany, during the conduct of the study; personal fees from Merck KGaA, Darmstadt, Germany, Ipsen, Lilly, and Versatis, outside the submitted work. DL is an employee of Merck KGaA, Darmstadt, Germany.

## References

[1] Maghnie M Labarta JI Koledova E Rohrer TR Short stature diagnosis and referral. Front Endocrinol (Lausanne) (2017). 10.3389/fendo.2017.00374 PMC5768898 29375479

[2] Hauffa BP Touraine P Urquhart T Koledova E Managing transition in patients treated with growth hormone. Front Endocrinol (Lausanne) (2017). 10.3389/fendo.2017.00346 PMC5732460 29312142

[3] Bang P Bjerknes R Dahlgren J Dunkel L Gustafsson J Juul A A comparison of different definitions of growth response in short prepubertal children treated with growth hormone. Horm Res Paediatr (2011) 75:335–45. 10.1159/000322878 21228552

[4] Savage MO Burren CP Rosenfeld RG. The continuum of growth hormone-IGF-I axis defects causing short stature: diagnostic and therapeutic challenges. Clin Endocrinol (Oxf) (2010) 72:721–8. 10.1111/j.1365-2265.2009.03775.x 20050859

[5] Bakker B Frane J Anhalt H Lippe B Rosenfeld RG. Height velocity targets from the national cooperative growth study for first-year growth hormone responses in short children. J Clin Endocrinol Metab (2008) 93:352–7. 10.1210/jc.2007-1581 18000092

[6] Ranke MB Lindberg A KIGS International Board. Observed and predicted growth responses in prepubertal children with growth disorders: guidance of growth hormone treatment by empirical variables. J Clin Endocrinol Metab (2010) 95:1229–37. 10.1210/jc.2009-1471 20097713

[7] Cohen P Germak J Rogol AD Weng W Kappelgaard AM Rosenfeld RG Variable degree of growth hormone (GH) and insulin-like growth factor (IGF) sensitivity in children with idiopathic short stature compared with GH-deficient patients: evidence from an IGF-based dosing study of short children. J Clin Endocrinol Metab (2010) 95:2089–98. 10.1210/jc.2009-2139 20207829

[8] Ranke MB Lindberg A Chatelain P Wilton P Price DA Albertsson-Wikland K The potential of prediction models based on data from KIGS as tools to measure responsiveness to growth hormone. Pharmacia International Growth Database. Horm Res (2001) 55(Suppl 2):44–8. 10.1159/000063474 11684876

[9] Kapoor RR Burke SA Sparrow SE Hughes IA Dunger DB Ong KK Monitoring of concordance in growth hormone therapy. Arch Dis Child (2008) 93:147–8. 10.1136/adc.2006.114249 17768149

[10] Cutfield WS Derraik JG Gunn AJ Reid K Delany T Robinson E Non-compliance with growth hormone treatment in children is common and impairs linear growth. PLoS One (2011) 6:e16223. 10.1371/journal.pone.0016223 21305004 PMC3031542

[11] Fisher BG Acerini CL. Understanding the growth hormone therapy adherence paradigm: a systematic review. Horm Res Paediatr (2013) 79:189–96. 10.1159/000350251 23635797

[12] Rosenfeld RG Bakker B. Compliance and persistence in pediatric and adult patients receiving growth hormone therapy. Endocr Pract (2008) 14:143–54. 10.4158/EP.14.2.143 18308651

[13] Michie S van Stralen MM West R. The behaviour change wheel: a new method for characterising and designing behaviour change interventions. Implement Sci (2011) 6:42. 10.1186/1748-5908-6-42 21513547 PMC3096582

[14] Jackson C Eliasson L Barber N Weinman J Applying COM-B to medication adherence. Eur Health Psychol (2014) 16:7–17.

[15] Godbout A Tejedor I Malivoir S Polak M Touraine P. Transition from pediatric to adult healthcare: assessment of specific needs of patients with chronic endocrine conditions. Horm Res Paediatr (2012) 78:247–55. 10.1159/000343818 23128858

[16] European Commission. Almost 8 Out of 10 Internet Users in the EU Surfed via a Mobile or Smart Phone in 2016 [Internet]. (2016). Available from: http://ec.europa.eu/eurostat/documents/2995521/7771139/9-20122016-BP-EN.pdf/f023d81a-dce2-4959-93e3-8cc7082b6edd

[17] Canalys. Over 17 Million Wearable Bands Are Forecast to Ship in 2014, Driven by Devices with Wearable-Specific Sensors [Internet]. (2014). Available from: https://canalys.com/newsroom/16-million-smart-bands-shipped-h2-2013

[18] Kruse W Rampmaier J Ullrich G Weber E. Patterns of drug compliance with medications to be taken once and twice daily assessed by continuous electronic monitoring in primary care. Int J Clin Pharmacol Ther (1994) 32:452–7. 7820327

[19] Cho JH Kim HS Yoo SH Jung CH Lee WJ Park CY An Internet-based health gateway device for interactive communication and automatic data uploading: clinical efficacy for type 2 diabetes in a multi-centre trial. J Telemed Telecare (2017) 23:595–604. 10.1177/1357633X16657500 27381040

[20] Clough BA Casey LM. Technological adjuncts to increase adherence to therapy: a review. Clin Psychol Rev (2011) 31:697–710. 10.1016/j.cpr.2011.03.006 21497153

[21] Cho JH Lee HC Lim DJ Kwon HS Yoon KH. Mobile communication using a mobile phone with a glucometer for glucose control in type 2 patients with diabetes: as effective as an Internet-based glucose monitoring system. J Telemed Telecare (2009) 15:77–82. 10.1258/jtt.2008.080412 19246607

[22] Vervloet M van Dijk L Santen-Reestman J van Vlijmen B van Wingerden P Bouvy ML SMS reminders improve adherence to oral medication in type 2 diabetes patients who are real time electronically monitored. Int J Med Inform (2012) 81:594–604. 10.1016/j.ijmedinf.2012.05.005 22652012

[23] Mbuagbaw L van der Kop ML Lester RT Thirumurthy H Pop-Eleches C Ye C Mobile phone text messages for improving adherence to antiretroviral therapy (ART): an individual patient data meta-analysis of randomised trials. BMJ Open (2013) 3:e003950 10.1136/bmjopen-2013-003950 PMC3884740 24345901

[24] Zurovac D Sudoi RK Akhwale WS Ndiritu M Hamer DH Rowe AK The effect of mobile phone text-message reminders on Kenyan health workers’ adherence to malaria treatment guidelines: a cluster randomised trial. Lancet (2011) 378:795–803. 10.1016/S0140-6736(11)60783-6 21820166 PMC3163847

[25] Mobihealthnews. Pillsy Launches Smart Pill Bottle and App to Improve Medication Adherence [Internet]. (2017). Available from: http://www.mobihealthnews.com/content/pillsy-launches-smart-pill-bottle-and-app-improve-medication-adherence

[26] Chan AH Stewart AW Harrison J Camargo CA Jr Black PN Mitchell EA. The effect of an electronic monitoring device with audiovisual reminder function on adherence to inhaled corticosteroids and school attendance in children with asthma: a randomised controlled trial. Lancet Respir Med (2015) 3:210–9. 10.1016/S2213-2600(15)00008-9 25617215

[27] Cassorla F Cianfarani S Haverkamp F Labarta JI Loche S Luo X Growth hormone and treatment outcomes: expert review of current clinical practice. Pediatr Endocrinol Rev (2011) 9:554–65. 22397140

[28] Ayala-Estrada A Antillon-Ferreira C Saavedra-Castillo E Barrientos-Pérez M Rivero-Escalante H Flores-Caloca O The easypod connect observational study (ECOS): descriptive analysis of adherence to treatment of growth hormone deficient and small for gestational age naïve to easypod patients in Mexico 2012-2015. Soc Latinoam Endocrinol Pediátr (2016):P–103.

[29] Dumas H Panayiotopoulos P Parker D Pongpairochana V. Understanding and meeting the needs of those using growth hormone injection devices. BMC Endocr Disord (2006) 6:5. 10.1186/1472-6823-6-5 17034628 PMC1618831

[30] Dahlgren J Veimo D Johansson L Bech I. Patient acceptance of a novel electronic auto-injector device to administer recombinant human growth hormone: results from an open-label, user survey of everyday use. Curr Med Res Opin (2007) 23:1649–55. 10.1185/030079907X210589 17559757

[31] Dahlgren J. Easypod: a new electronic injection device for growth hormone. Expert Rev Med Devices (2008) 5:297–304. 10.1586/17434440.5.3.297 18452378

[32] Bozzola M Colle M Halldin-Stenlid M Larroque S Zignani M Easypod™ Survey Study Group Treatment adherence with the easypod™ growth hormone electronic auto-injector and patient acceptance: survey results from 824 children and their parents. BMC Endocr Disord (2011) 11:4 10.1186/1472-6823-11-4 21294891 PMC3045978

[33] Davies P Nicolino M Norgren S Stoyanov G Koledova E VanderMeulen J The easypod™ connect observational study: comparison of results from interim analyses. Horm Res Pediatr (2015) 84(Suppl 1):464.

[34] Broens TH Huis in’t Veld RM Vollenbroek-Hutten MM Hermens HJ van Halteren AT Nieuwenhuis LJ. Determinants of successful telemedicine implementations: a literature study. J Telemed Telecare (2007) 13:303–9. 10.1258/135763307781644951 17785027

[35] Tauber M De Bouet Du Portal H Sallerin-Caute B Rochiccioli P Bastide R. Differential regulation of serum growth hormone (GH)-binding protein during continuous infusion versus daily injection of recombinant human GH in GH-deficient children. J Clin Endocrinol Metab (1993) 76:1135–9. 10.1210/jcem.76.5.8496303 8496303

[36] Christiansen JS Backeljauw PF Bidlingmaier M Biller BM Boguszewski MC Casanueva FF Growth hormone research society perspective on the development of long-acting growth hormone preparations. Eur J Endocrinol (2016) 174:C1–8. 10.1530/EJE-16-0111 27009113 PMC5081743

[37] Høybye C Cohen P Hoffman AR Ross R Biller BM Christiansen JS Status of long-acting-growth hormone preparations—2015. Growth Horm IGF Res (2015) 25:201–6. 26187188 10.1016/j.ghir.2015.07.004

[38] Loche S Salerno M Garofalo P Cardinale GM Licenziati MR Citro G Adherence in children with growth hormone deficiency treated with r-hGH and the easypod™ device. J Endocrinol Invest (2016) 39:1419–24. 10.1007/s40618-016-0510-0 27406716 PMC5107197

[39] Deacon AJ Edirippulige S. Using mobile technology to motivate adolescents with type 1 diabetes mellitus: a systematic review of recent literature. J Telemed Telecare (2015) 21:431–8. 10.1177/1357633X15605223 26377124

[40] Galea MN Weinman JA White C Bearne LM. Do behaviour-change techniques contribute to the effectiveness of exercise therapy in patients with intermittent claudication? A systematic review. Eur J Vasc Endovasc Surg (2013) 46:132–41. 10.1016/j.ejvs.2013.03.030 23664887 PMC4033840

